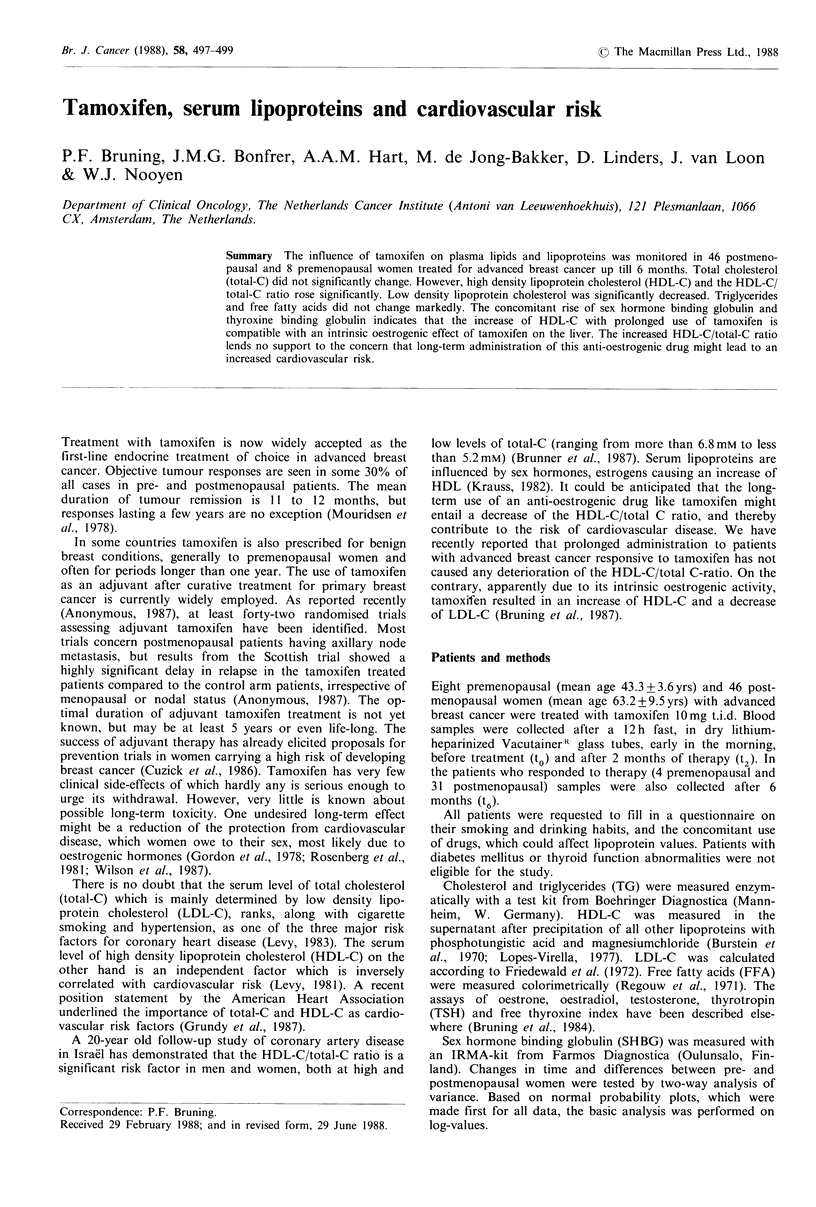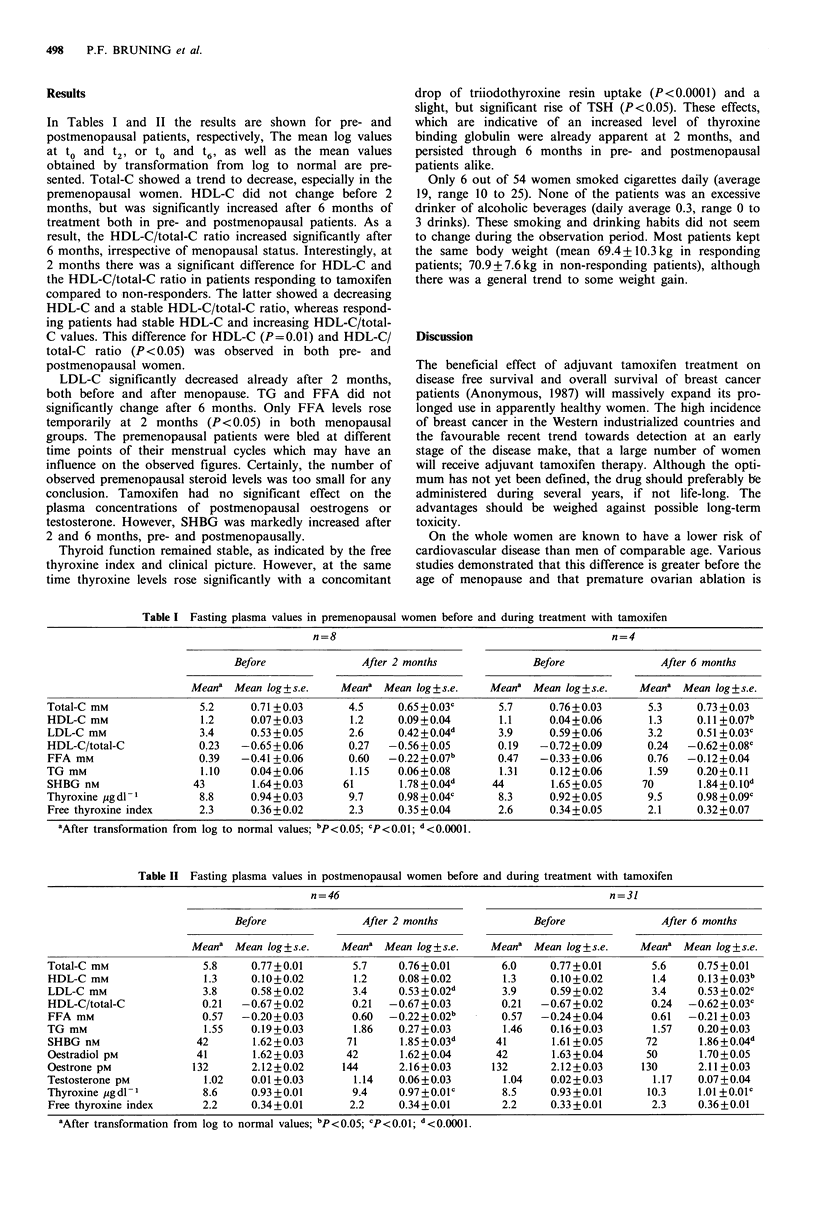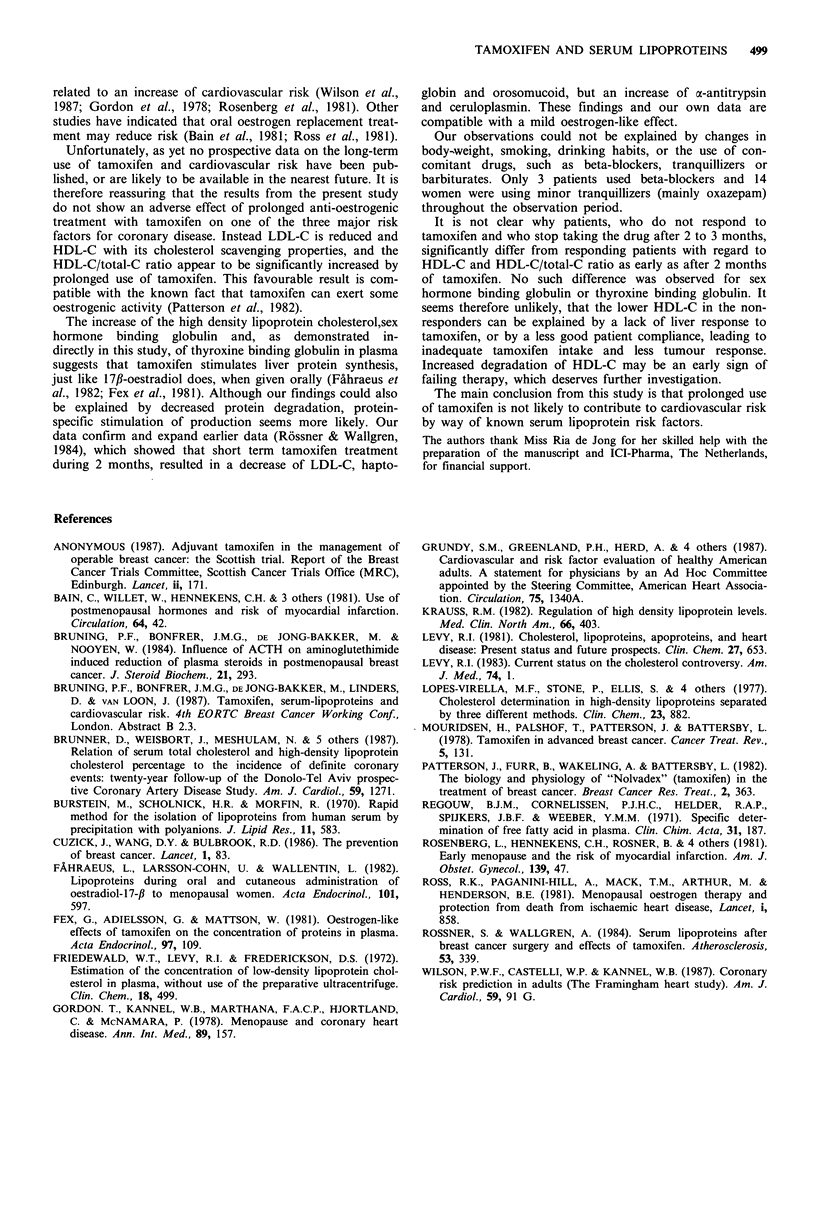# Tamoxifen, serum lipoproteins and cardiovascular risk.

**DOI:** 10.1038/bjc.1988.248

**Published:** 1988-10

**Authors:** P. F. Bruning, J. M. Bonfrer, A. A. Hart, M. de Jong-Bakker, D. Linders, J. van Loon, W. J. Nooyen

**Affiliations:** Department of Clinical Oncology, The Netherlands Cancer Institute, Amsterdam.

## Abstract

The influence of tamoxifen on plasma lipids and lipoproteins was monitored in 46 postmenopausal and 8 premenopausal women treated for advanced breast cancer up till 6 months. Total cholesterol (total-C) did not significantly change. However, high density lipoprotein cholesterol (HDL-C) and the HDL-C/total-C ratio rose significantly. Low density lipoprotein cholesterol was significantly decreased. Triglycerides and free fatty acids did not change markedly. The concomitant rise of sex hormone binding globulin and thyroxine binding globulin indicates that the increase of HDL-C with prolonged use of tamoxifen is compatible with an intrinsic oestrogenic effect of tamoxifen on the liver. The increased HDL-C/total-C ratio lends no support to the concern that long-term administration of this anti-oestrogenic drug might lead to an increased cardiovascular risk.


*Br. J. Cancer* (1988), **58**, 497–499 © The Macmillan Press Ltd., 1988

### Tamoxifen, serum lipoproteins and cardiovascular risk

P.F. Bruning, J.M.G. Bonfrer, A.A.M. Hart, M. de Jong-Bakker, D. Linders, J. van Loon & W.J. Nooyen

*Department of Clinical Oncology, The Netherlands Cancer Institute (Antoni van Leeuwenhoekhuis), 121 Plesmanlaan, 1066 CX, Amsterdam, The Netherlands.*

**Summary** The influence of tamoxifen on plasma lipids and lipoproteins was monitored in 46 postmenopausal and 8 premenopausal women treated for advanced breast cancer up till 6 months. Total cholesterol (total-C) did not significantly change. However, high density lipoprotein cholesterol (HDL-C) and the HDL-C/total-C ratio rose significantly. Low density lipoprotein cholesterol was significantly decreased. Triglycerides and free fatty acids did not change markedly. The concomitant rise of sex hormone binding globulin and thyroxine binding globulin indicates that the increase of HDL-C with prolonged use of tamoxifen is compatible with an intrinsic oestrogenic effect of tamoxifen on the liver. The increased HDL-C/total-C ratio lends no support to the concern that long-term administration of this anti-oestrogenic drug might lead to an increased cardiovascular risk.

Treatment with tamoxifen is now widely accepted as the first-line endocrine treatment of choice in advanced breast cancer. Objective tumour responses are seen in some 30% of all cases in pre- and postmenopausal patients. The mean duration of tumour remission is 11 to 12 months, but responses lasting a few years are no exception (Mouridsen *et al.*, 1978).

In some countries tamoxifen is also prescribed for benign breast conditions, generally to premenopausal women and often for periods longer than one year. The use of tamoxifen as an adjuvant after curative treatment for primary breast cancer is currently widely employed. As reported recently (Anonymous, 1987), at least forty-two randomised trials assessing adjuvant tamoxifen have been identified. Most trials concern postmenopausal patients having axillary node metastasis, but results from the Scottish trial showed a highly significant delay in relapse in the tamoxifen treated patients compared to the control arm patients, irrespective of menopausal or nodal status (Anonymous, 1987). The optimal duration of adjuvant tamoxifen treatment is not yet known, but may be at least 5 years or even life-long. The success of adjuvant therapy has already elicited proposals for prevention trials in women carrying a high risk of developing breast cancer (Cuzick *et al.*, 1986). Tamoxifen has very few clinical side-effects of which hardly any is serious enough to urge its withdrawal. However, very little is known about possible long-term toxicity. One undesired long-term effect might be a reduction of the protection from cardiovascular disease, which women owe to their sex, most likely due to oestrogenic hormones (Gordon *et al.*, 1978; Rosenberg *et al.*, 1981; Wilson *et al.*, 1987).

There is no doubt that the serum level of total cholesterol (total-C) which is mainly determined by low density lipoprotein cholesterol (LDL-C), ranks, along with cigarette smoking and hypertension, as one of the three major risk factors for coronary heart disease (Levy, 1983). The serum level of high density lipoprotein cholesterol (HDL-C) on the other hand is an independent factor which is inversely correlated with cardiovascular risk (Levy, 1981). A recent position statement by the American Heart Association underlined the importance of total-C and HDL-C as cardiovascular risk factors (Grundy *et al.*, 1987).

A 20-year old follow-up study of coronary artery disease in Israël has demonstrated that the HDL-C/total-C ratio is a significant risk factor in men and women, both at high and low levels of total-C (ranging from more than 6.8 mM to less than 5.2 mM) (Brunner *et al.*, 1987). Serum lipoproteins are influenced by sex hormones, estrogens causing an increase of HDL (Krauss, 1982). It could be anticipated that the long-term use of an anti-oestrogenic drug like tamoxifen might entail a decrease of the HDL-C/total C ratio, and thereby contribute to the risk of cardiovascular disease. We have recently reported that prolonged administration to patients with advanced breast cancer responsive to tamoxifen has not caused any deterioration of the HDL-C/total C-ratio. On the contrary, apparently due to its intrinsic oestrogenic activity, tamoxifen resulted in an increase of HDL-C and a decrease of LDL-C (Bruning *et al.*, 1987).

### Patients and methods

Eight premenopausal (mean age 43.3 ± 3.6 yrs) and 46 postmenopausal women (mean age 63.2 ± 9.5 yrs) with advanced breast cancer were treated with tamoxifen 10 mg t.i.d. Blood samples were collected after a 12 h fast, in dry lithium-heparinized Vacutainer® glass tubes, early in the morning, before treatment ($t_0$) and after 2 months of therapy ($t_2$). In the patients who responded to therapy (4 premenopausal and 31 postmenopausal) samples were also collected after 6 months ($t_6$).

All patients were requested to fill in a questionnaire on their smoking and drinking habits, and the concomitant use of drugs, which could affect lipoprotein values. Patients with diabetes mellitus or thyroid function abnormalities were not eligible for the study.

Cholesterol and triglycerides (TG) were measured enzymatically with a test kit from Boehringer Diagnostica (Mannheim, W. Germany). HDL-C was measured in the supernatant after precipitation of all other lipoproteins with phosphotungistic acid and magnesiumchloride (Burstein *et al.*, 1970; Lopes-Virella, 1977). LDL-C was calculated according to Friedewald *et al.* (1972). Free fatty acids (FFA) were measured colorimetrically (Regouw *et al.*, 1971). The assays of oestrone, oestradiol, testosterone, thyrotropin (TSH) and free thyroxine index have been described elsewhere (Bruning *et al.*, 1984).

Sex hormone binding globulin (SHBG) was measured with an IRMA-kit from Farmos Diagnostica (Oulunsalo, Finland). Changes in time and differences between pre- and postmenopausal women were tested by two-way analysis of variance. Based on normal probability plots, which were made first for all data, the basic analysis was performed on log-values.

Correspondence: P.F. Bruning.
Received 29 February 1988; and in revised form, 29 June 1988.

### Results

In Tables I and II the results are shown for pre- and postmenopausal patients, respectively, The mean log values at $t_0$ and $t_2$, or $t_0$ and $t_6$, as well as the mean values obtained by transformation from log to normal are presented. Total-C showed a trend to decrease, especially in the premenopausal women. HDL-C did not change before 2 months, but was significantly increased after 6 months of treatment both in pre- and postmenopausal patients. As a result, the HDL-C/total-C ratio increased significantly after 6 months, irrespective of menopausal status. Interestingly, at 2 months there was a significant difference for HDL-C and the HDL-C/total-C ratio in patients responding to tamoxifen compared to non-responders. The latter showed a decreasing HDL-C and a stable HDL-C/total-C ratio, whereas responding patients had stable HDL-C and increasing HDL-C/total-C values. This difference for HDL-C ($P=0.01$) and HDL-C/total-C ratio ($P<0.05$) was observed in both pre- and postmenopausal women.

LDL-C significantly decreased already after 2 months, both before and after menopause. TG and FFA did not significantly change after 6 months. Only FFA levels rose temporarily at 2 months ($P<0.05$) in both menopausal groups. The premenopausal patients were bled at different time points of their menstrual cycles which may have an influence on the observed figures. Certainly, the number of observed premenopausal steroid levels was too small for any conclusion. Tamoxifen had no significant effect on the plasma concentrations of postmenopausal oestrogens or testosterone. However, SHBG was markedly increased after 2 and 6 months, pre- and postmenopausally.

Thyroid function remained stable, as indicated by the free thyroxine index and clinical picture. However, at the same time thyroxine levels rose significantly with a concomitant drop of triiodothyroxine resin uptake ($P<0.0001$) and a slight, but significant rise of TSH ($P<0.05$). These effects, which are indicative of an increased level of thyroxine binding globulin were already apparent at 2 months, and persisted through 6 months in pre- and postmenopausal patients alike.

Only 6 out of 54 women smoked cigarettes daily (average 19, range 10 to 25). None of the patients was an excessive drinker of alcoholic beverages (daily average 0.3, range 0 to 3 drinks). These smoking and drinking habits did not seem to change during the observation period. Most patients kept the same body weight (mean 69.4±10.3 kg in responding patients; 70.9±7.6 kg in non-responding patients), although there was a general trend to some weight gain.

### Discussion

The beneficial effect of adjuvant tamoxifen treatment on disease free survival and overall survival of breast cancer patients (Anonymous, 1987) will massively expand its prolonged use in apparently healthy women. The high incidence of breast cancer in the Western industrialized countries and the favourable recent trend towards detection at an early stage of the disease make, that a large number of women will receive adjuvant tamoxifen therapy. Although the optimum has not yet been defined, the drug should preferably be administered during several years, if not life-long. The advantages should be weighed against possible long-term toxicity.

On the whole women are known to have a lower risk of cardiovascular disease than men of comparable age. Various studies demonstrated that this difference is greater before the age of menopause and that premature ovarian ablation is

**Table I** Fasting plasma values in premenopausal women before and during treatment with tamoxifen

| | *n=8* | | | | *n=4* | | | |
|---|---|---|---|---|---|---|---|---|
| | *Before* | | *After 2 months* | | *Before* | | *After 6 months* | |
| | *Mean*[a] | *Mean log±s.e.* | *Mean*[a] | *Mean log±s.e.* | *Mean*[a] | *Mean log±s.e.* | *Mean*[a] | *Mean log±s.e.* |
| Total-C mM | 5.2 | 0.71±0.03 | 4.5 | 0.65±0.03[c] | 5.7 | 0.76±0.03 | 5.3 | 0.73±0.03 |
| HDL-C mM | 1.2 | 0.07±0.03 | 1.2 | 0.09±0.04 | 1.1 | 0.04±0.06 | 1.3 | 0.11±0.07[b] |
| LDL-C mM | 3.4 | 0.53±0.05 | 2.6 | 0.42±0.04[d] | 3.9 | 0.59±0.06 | 3.2 | 0.51±0.03[c] |
| HDL-C/total-C | 0.23 | −0.65±0.06 | 0.27 | −0.56±0.05 | 0.19 | −0.72±0.09 | 0.24 | −0.62±0.08[c] |
| FFA mM | 0.39 | −0.41±0.06 | 0.60 | −0.22±0.07[b] | 0.47 | −0.33±0.06 | 0.76 | −0.12±0.04 |
| TG mM | 1.10 | 0.04±0.06 | 1.15 | 0.06±0.08 | 1.31 | 0.12±0.06 | 1.59 | 0.20±0.11 |
| SHBG nM | 43 | 1.64±0.03 | 61 | 1.78±0.04[d] | 44 | 1.65±0.05 | 70 | 1.84±0.10[d] |
| Thyroxine μg dl$^{-1}$ | 8.8 | 0.94±0.03 | 9.7 | 0.98±0.04[c] | 8.3 | 0.92±0.05 | 9.5 | 0.98±0.09[c] |
| Free thyroxine index | 2.3 | 0.36±0.02 | 2.3 | 0.35±0.04 | 2.6 | 0.34±0.05 | 2.1 | 0.32±0.07 |

[a]After transformation from log to normal values; [b]$P<0.05$; [c]$P<0.01$; [d]$<0.0001$.

**Table II** Fasting plasma values in postmenopausal women before and during treatment with tamoxifen

| | *n=46* | | | | *n=31* | | | |
|---|---|---|---|---|---|---|---|---|
| | *Before* | | *After 2 months* | | *Before* | | *After 6 months* | |
| | *Mean*[a] | *Mean log±s.e.* | *Mean*[a] | *Mean log±s.e.* | *Mean*[a] | *Mean log±s.e.* | *Mean*[a] | *Mean log±s.e.* |
| Total-C mM | 5.8 | 0.77±0.01 | 5.7 | 0.76±0.01 | 6.0 | 0.77±0.01 | 5.6 | 0.75±0.01 |
| HDL-C mM | 1.3 | 0.10±0.02 | 1.2 | 0.08±0.02 | 1.3 | 0.10±0.02 | 1.4 | 0.13±0.03[b] |
| LDL-C mM | 3.8 | 0.58±0.02 | 3.4 | 0.53±0.02[d] | 3.9 | 0.59±0.02 | 3.4 | 0.53±0.02[c] |
| HDL-C/total-C | 0.21 | −0.67±0.02 | 0.21 | −0.67±0.03 | 0.21 | −0.67±0.02 | 0.24 | −0.62±0.03[c] |
| FFA mM | 0.57 | −0.20±0.03 | 0.60 | −0.22±0.02[b] | 0.57 | −0.24±0.04 | 0.61 | −0.21±0.03 |
| TG mM | 1.55 | 0.19±0.03 | 1.86 | 0.27±0.03 | 1.46 | 0.16±0.03 | 1.57 | 0.20±0.03 |
| SHBG nM | 42 | 1.62±0.03 | 71 | 1.85±0.03[d] | 41 | 1.61±0.05 | 72 | 1.86±0.04[d] |
| Oestradiol pM | 41 | 1.62±0.03 | 42 | 1.62±0.04 | 42 | 1.63±0.04 | 50 | 1.70±0.05 |
| Oestrone pM | 132 | 2.12±0.02 | 144 | 2.16±0.03 | 132 | 2.12±0.03 | 130 | 2.11±0.03 |
| Testosterone pM | 1.02 | 0.01±0.03 | 1.14 | 0.06±0.03 | 1.04 | 0.02±0.03 | 1.17 | 0.07±0.04 |
| Thyroxine μg dl$^{-1}$ | 8.6 | 0.93±0.01 | 9.4 | 0.97±0.01[c] | 8.5 | 0.93±0.01 | 10.3 | 1.01±0.01[c] |
| Free thyroxine index | 2.2 | 0.34±0.01 | 2.2 | 0.34±0.01 | 2.2 | 0.33±0.01 | 2.3 | 0.36±0.01 |

[a]After transformation from log to normal values; [b]$P<0.05$; [c]$P<0.01$; [d]$<0.0001$.

related to an increase of cardiovascular risk (Wilson *et al.*, 1987; Gordon *et al.*, 1978; Rosenberg *et al.*, 1981). Other studies have indicated that oral oestrogen replacement treatment may reduce risk (Bain *et al.*, 1981; Ross *et al.*, 1981).

Unfortunately, as yet no prospective data on the long-term use of tamoxifen and cardiovascular risk have been published, or are likely to be available in the nearest future. It is therefore reassuring that the results from the present study do not show an adverse effect of prolonged anti-oestrogenic treatment with tamoxifen on one of the three major risk factors for coronary disease. Instead LDL-C is reduced and HDL-C with its cholesterol scavenging properties, and the HDL-C/total-C ratio appear to be significantly increased by prolonged use of tamoxifen. This favourable result is compatible with the known fact that tamoxifen can exert some oestrogenic activity (Patterson *et al.*, 1982).

The increase of the high density lipoprotein cholesterol,sex hormone binding globulin and, as demonstrated indirectly in this study, of thyroxine binding globulin in plasma suggests that tamoxifen stimulates liver protein synthesis, just like 17$\beta$-oestradiol does, when given orally (Fåhraeus *et al.*, 1982; Fex *et al.*, 1981). Although our findings could also be explained by decreased protein degradation, protein-specific stimulation of production seems more likely. Our data confirm and expand earlier data (Rössner & Wallgren, 1984), which showed that short term tamoxifen treatment during 2 months, resulted in a decrease of LDL-C, haptoglobin and orosomucoid, but an increase of $\alpha$-antitrypsin and ceruloplasmin. These findings and our own data are compatible with a mild oestrogen-like effect.

Our observations could not be explained by changes in body-weight, smoking, drinking habits, or the use of concomitant drugs, such as beta-blockers, tranquillizers or barbiturates. Only 3 patients used beta-blockers and 14 women were using minor tranquillizers (mainly oxazepam) throughout the observation period.

It is not clear why patients, who do not respond to tamoxifen and who stop taking the drug after 2 to 3 months, significantly differ from responding patients with regard to HDL-C and HDL-C/total-C ratio as early as after 2 months of tamoxifen. No such difference was observed for sex hormone binding globulin or thyroxine binding globulin. It seems therefore unlikely, that the lower HDL-C in the non-responders can be explained by a lack of liver response to tamoxifen, or by a less good patient compliance, leading to inadequate tamoxifen intake and less tumour response. Increased degradation of HDL-C may be an early sign of failing therapy, which deserves further investigation.

The main conclusion from this study is that prolonged use of tamoxifen is not likely to contribute to cardiovascular risk by way of known serum lipoprotein risk factors.

The authors thank Miss Ria de Jong for her skilled help with the preparation of the manuscript and ICI-Pharma, The Netherlands, for financial support.

### References

ANONYMOUS (1987). Adjuvant tamoxifen in the management of operable breast cancer: the Scottish trial. Report of the Breast Cancer Trials Committee, Scottish Cancer Trials Office (MRC), Edinburgh. *Lancet*, **ii**, 171.

BAIN, C., WILLET, W., HENNEKENS, C.H. & 3 others (1981). Use of postmenopausal hormones and risk of myocardial infarction. *Circulation*, **64**, 42.

BRUNING, P.F., BONFRER, J.M.G., DE JONG-BAKKER, M. & NOOYEN, W. (1984). Influence of ACTH on aminoglutethimide induced reduction of plasma steroids in postmenopausal breast cancer. *J. Steroid Biochem.*, **21**, 293.

BRUNING, P.F., BONFRER, J.M.G., DE JONG-BAKKER, M., LINDERS, D. & VAN LOON, J. (1987). Tamoxifen, serum-lipoproteins and cardiovascular risk. *4th EORTC Breast Cancer Working Conf.*, London. Abstract B 2.3.

BRUNNER, D., WEISBORT, J., MESHULAM, N. & 5 others (1987). Relation of serum total cholesterol and high-density lipoprotein cholesterol percentage to the incidence of definite coronary events: twenty-year follow-up of the Donolo-Tel Aviv prospective Coronary Artery Disease Study. *Am. J. Cardiol.*, **59**, 1271.

BURSTEIN, M., SCHOLNICK, H.R. & MORFIN, R. (1970). Rapid method for the isolation of lipoproteins from human serum by precipitation with polyanions. *J. Lipid Res.*, **11**, 583.

CUZICK, J., WANG, D.Y. & BULBROOK, R.D. (1986). The prevention of breast cancer. *Lancet*, **1**, 83.

FÅHRAEUS, L., LARSSON-COHN, U. & WALLENTIN, L. (1982). Lipoproteins during oral and cutaneous administration of oestradiol-17-$\beta$ to menopausal women. *Acta Endocrinol.*, **101**, 597.

FEX, G., ADIELSSON, G. & MATTSON, W. (1981). Oestrogen-like effects of tamoxifen on the concentration of proteins in plasma. *Acta Endocrinol.*, **97**, 109.

FRIEDEWALD, W.T., LEVY, R.I. & FREDERICKSON, D.S. (1972). Estimation of the concentration of low-density lipoprotein cholesterol in plasma, without use of the preparative ultracentrifuge. *Clin. Chem.*, **18**, 499.

GORDON. T., KANNEL, W.B., MARTHANA, F.A.C.P., HJORTLAND, C. & MCNAMARA, P. (1978). Menopause and coronary heart disease. *Ann. Int. Med.*, **89**, 157.

GRUNDY, S.M., GREENLAND, P.H., HERD, A. & 4 others (1987). Cardiovascular and risk factor evaluation of healthy American adults. A statement for physicians by an Ad Hoc Committee appointed by the Steering Committee, American Heart Association. *Circulation*, **75**, 1340A.

KRAUSS, R.M. (1982). Regulation of high density lipoprotein levels. *Med. Clin. North Am.*, **66**, 403.

LEVY, R.I. (1981). Cholesterol, lipoproteins, apoproteins, and heart disease: Present status and future prospects. *Clin. Chem.* **27**, 653.

LEVY, R.I. (1983). Current status on the cholesterol controversy. *Am. J. Med.*, **74**, 1.

LOPES-VIRELLA, M.F., STONE, P., ELLIS, S. & 4 others (1977). Cholesterol determination in high-density lipoproteins separated by three different methods. *Clin. Chem.*, **23**, 882.

MOURIDSEN, H., PALSHOF, T., PATTERSON, J. & BATTERSBY, L. (1978). Tamoxifen in advanced breast cancer. *Cancer Treat. Rev.*, **5**, 131.

PATTERSON, J., FURR, B., WAKELING, A. & BATTERSBY, L. (1982). The biology and physiology of "Nolvadex" (tamoxifen) in the treatment of breast cancer. *Breast Cancer Res. Treat.*, **2**, 363.

REGOUW, B.J.M., CORNELISSEN, P.J.H.C., HELDER, R.A.P., SPIJKERS, J.B.F. & WEEBER, Y.M.M. (1971). Specific determination of free fatty acid in plasma. *Clin. Chim. Acta*, **31**, 187.

ROSENBERG, L., HENNEKENS, C.H., ROSNER, B. & 4 others (1981). Early menopause and the risk of myocardial infarction. *Am. J. Obstet. Gynecol.*, **139**, 47.

ROSS, R.K., PAGANINI-HILL, A., MACK, T.M., ARTHUR, M. & HENDERSON, B.E. (1981). Menopausal oestrogen therapy and protection from death from ischaemic heart disease, *Lancet*, **i**, 858.

ROSSNER, S. & WALLGREN, A. (1984). Serum lipoproteins after breast cancer surgery and effects of tamoxifen. *Atherosclerosis*, **53**, 339.

WILSON, P.W.F., CASTELLI, W.P. & KANNEL, W.B. (1987). Coronary risk prediction in adults (The Framingham heart study). *Am. J. Cardiol.*, **59**, 91 G.